# Effects of Nutrition/Diet on Brown Adipose Tissue in Humans: A Systematic Review and Meta-Analysis

**DOI:** 10.3390/nu12092752

**Published:** 2020-09-10

**Authors:** Kelsey A. Heenan, Andres E. Carrillo, Jacob L. Fulton, Edward J. Ryan, Jason R. Edsall, Dimitrios Rigopoulos, Melissa M. Markofski, Andreas D. Flouris, Petros C. Dinas

**Affiliations:** 1 Department of Movement Science, Chatham University, Pittsburgh, PA 15232, USA; Kelsey.Heenan@chatham.edu (K.A.H.); aecarrill@gmail.com (A.E.C.); jacob.fulton04@gmail.com (J.L.F.); ERyan@chatham.edu (E.J.R.); J.Edsall@chatham.edu (J.R.E.); 2 FAME Laboratory, Department of Exercise Science, University of Thessaly, GR42100 Trikala, Greece; dr.rigopoulos@gmail.com (D.R.); andreasflouris@gmail.com (A.D.F.); 3 Department of Health and Human Performance, University of Houston, Houston, TX 77204, USA; mmarkofs@central.uh.edu

**Keywords:** BAT, diet-induced thermogenesis, thermic effect of food

## Abstract

Background: Brown adipose tissue (BAT) provides a minor contribution to diet-induced thermogenesis (DIT)—the metabolic response to food consumption. Increased BAT activity is generally considered beneficial for mammalian metabolism and has been associated with favorable health outcomes. The aim of the current systematic review was to explore whether nutritional factors and/or diet affect human BAT activity. Methods: We searched PubMed Central, Embase and Cochrane Library (trials) to conduct this systematic review (PROSPERO protocol: CRD42018082323). Results: We included 24 eligible papers that studied a total of 2785 participants. We found no mean differences in standardized uptake value of BAT following a single meal or after 6 weeks of L-Arginine supplementation. Resting energy expenditure (REE), however, was increased following a single meal and after supplementation of capsinoid and catechin when compared to a control condition (Z = 2.41, *p* = 0.02; mean difference = 102.47 (95% CI = 19.28–185.67)). Conclusions: Human BAT activity was not significantly affected by nutrition/diet. Moreover, REE was only increased in response to a single meal, but it is unlikely that this was due to increased BAT activity. BAT activity assessments in response to the chronic effect of food should be considered along with other factors such as body composition and/or environmental temperature.

## 1. Introduction

Brown adipose tissue (BAT) is located in the lower neck, collarbone, abdomen, and along the spine in humans [1]. It is activated through the release of norepinephrine from the sympathetic nervous system that triggers adrenergic receptors on the surface of brown adipocytes [2]. Subsequently, uncoupling protein one (UCP1) utilizes potential energy created by a proton gradient to generate heat rather than adenosine triphosphate (ATP) [2]. This phenomenon occurs during (1) non-shivering thermogenesis characterized by heat production during cold exposure [2], or (2) as a minor contribution to diet-induced thermogenesis (DIT) [3,4]. DIT can be described as the metabolic response to food intake with the majority of the energy cost due to the digestion and metabolism of ingested nutrients [5].

BAT activity is considered beneficial for mammalian metabolism, given that in animals it reduces weight gain, improves glucose tolerance and insulin sensitivity, decreases the risk for type 2 diabetes, lowers free fatty acid levels in serum, reduces elevated triglycerides, decreases hypercholesterolaemia, provides protection from the development of atherosclerosis and improves other metabolic conditions [6,7,8,9,10,11,12,13,14]. In humans, BAT activity is generally lower in overweight and obese males when compared to lean healthy males [1]. Further, it is more active in young than older individuals and also more active in men than women [15]. Indeed, body mass index (BMI) [15], age [16] and fat mass [1] are inversely associated with BAT activity. This is not surprising, since BMI and fat mass are inversely associated with white adipose tissue thermogenic activity in humans [17].

Considering the beneficial effects of BAT activity on mammalian health, the possible role of diet in the chronic regulation of BAT activity is intriguing given the potential for improved metabolic health. For example, rats on a diet low in protein and high in carbohydrate increased resting energy expenditure (REE) via BAT activation [18,19]. Additionally, rodents fed high-fat diets may enhance cold tolerance and survival [20,21,22], as well as increase mitochondria in BAT [23]. These results may be in part explained by evidence that mice fed a ketogenic diet (i.e., high-fat, adequate-protein and low-carbohydrate) can increase the total number of mitochondria in BAT and UCP1 expression by two-fold [24,25], while increasing UCP1 expression in epididymal white adipose tissue [24]. UCP1 may enhance DIT in mouse BAT, while its absence may increase obesity in mice fed with a high-fat or typical diet [26]. It should be noted that DIT is entirely UCP1-dependent in mice [27,28].

To date, evidence in humans describing the effects of nutrition/diet on BAT and DIT is limited and inconsistent. For example, postprandial standardized uptake value (SUV) in BAT—measured with Positron Emission Tomography/Computed Tomography (PET/CT)—assessed in lean, healthy young males was less pronounced than when compared to a fasted state [29]. In another study, BAT positive (SUV > 2) participants displayed higher DIT and fat utilization compared to BAT negative (SUV < 2) participants [30]. A single, carbohydrate-rich meal increased glucose uptake in BAT among lean, healthy males [31], while Peterson et al. [32] reported that males who were overfed by 40% for 8 weeks experienced no change in BAT activity. This evidence indicates that a comprehensive investigation on the effects of nutrition/diet on human BAT activity is warranted. Therefore, the aim of the current systematic review was to explore whether nutritional factors and/or diet affects human BAT activity. As BAT activity represents a minor component of DIT [3], we also considered publications that examined DIT to explain the thermogenic effect of food in humans.

## 2. Materials and Methods

The current systematic review was registered with PROSPERO (review protocol: CRD42018082323).

### 2.1. Search Strategy and Selection Criteria

Following the Preferred Reporting Items for Systematic Reviews and Meta-analyses (PRISMA) guidelines [33], two independent investigators (PCD and KH) searched the PubMed Central, Embase and Cochrane Library (trials) databases from the date of their inception to 28 April 2020, for studies that evaluated the effects of nutrition/diet on BAT activity in humans. No date, participants’ health status, language or study design limits were applied. The searching algorithm can be found in Appendix A. We also searched the reference lists of the included studies in the systematic review, to identify potential eligible studies that were not included in the initial database searches. Disagreements in searching procedure between the two independent investigators were resolved through consensus.

Two independent investigators (KH and JLF) selected the eligible publications to be included in the systematic review. Conflicts in the selection of an eligible publication were resolved through a referee investigator (AEC). We included experimental studies that examined the effects of any nutritional element or diet on human BAT and on human DIT, while we excluded reviews, editorials, letters, magazine articles and conference proceedings.

### 2.2. Data Extraction and Quality Assessment

Two investigators (KH and DR) performed independent data extraction and conflicts were resolved through consensus and supervision by a third researcher (AEC). For all studies, we extracted the author name(s), year of publication, data on the participants’ number, age, sex, BMI and data on nutrition/diet intervention. Finally, we extracted a main outcome of nutrition/diet intervention on BAT activity/volume and DIT measured via REE. The extracted data can be found in Appendix A.

Two independent investigators (KH and DR) evaluated the risk of bias of the included studies in the systematic review. Conflicts in the risk of bias assessment were resolved by an independent referee investigator (PCD). For the eligible randomized controlled trials (RCT), we used the Cochrane Library tool for risk of bias assessment [34], while for the eligible observational design studies, we evaluated the risk of bias via the 13-item of Research Triangle Institute item bank [35], which is designed for observational studies and has previously shown median interrater agreement of 75% [36] and 93.5% [37].

### 2.3. Data Synthesis and Meta-Analysis Methods

For the studies that did not provide suitable data for a meta-analysis, a narrative data synthesis was used. For the studies that provided suitable data for a meta-analysis, we conducted a continuous, inverse variance, random-effect model meta-analysis via the RevMan 5.3 software [38]. We used means and standard deviations to test differences in BAT activity (SUV) and REE (kcal/day) between participants who undertook a dietary intervention against participants who did not undertake a dietary intervention or were fasted. Mean differences between a diet and a non-diet intervention were tested either pre and post diet or between a diet group and a control (non-diet) group. Moreover, the effects of diet on SUV and REE were tested for both acute and chronic interventions. The study effect sizes were synthesized to account for heterogeneity due to differences in study populations, interventions, study duration, and other factors. We evaluated the 95% confidence interval (CI) and heterogeneity between studies using the I² statistic. We considered a statistically significant result for heterogeneity when *p* < 0.10, while interpretation of I^2^ index was made based on previous guidelines [39]. Where pertinent, standard error (SE) was converted to standard deviation (SD) using the following formula: SD = SE ×√n [39].

## 3. Results

The reporting information of the current systematic review can be found in a relevant PRISMA checklist provided in the Appendix A.

### 3.1. Searching and Selection Procedure Results

The database search process identified 1847 publications, of which 452 were duplicates. We excluded 500 reviews, editorials, magazines and conference proceedings. Of the 895 remaining publications, 882 classified as non-eligible. As such, 13 publications met the inclusion criteria. We also supplemented our eligible publications with one paper that was added manually and 10 papers that were identified as eligible through reference lists screening of the 13 publications that initially met the inclusion criteria. Overall, 24 publications were included in the systematic review. The searching procedure results are illustrated in a PRISMA flowchart provided in Appendix A.

### 3.2. Characteristics of the Included Studies

The 24 included studies in the systematic review were published between 1984 and 2019 and involved 2785 participants. Nine of the included studies were RCTs, three controlled trials (CT), eight single group design studies (SGDS) and four cross-sectional studies (CSS). One RCT [40] and one CSS [41] examined the effects/associations of dietary factors on BAT activity through measurements of core and skin temperatures. Five RCTs [29,42,43,44,45], one CSS [46], one CT [47] and one SGDS [31] studied the effects/associations of dietary elements on BAT activity using PET/CT, while a SGDS [32] used infrared camera for BAT assessments. One SGDS [48] and one CSS [49] examined the effects/associations of dietary factors on BAT activity via measurements of UCP1. Finally, three RCTs [50,51,52], two CTs [53,54], five SGDS [55,56,57,58,59] and one CSS [30] indirectly assessed BAT activity via DIT. The characteristics of the included studies are found in the Appendix A.

### 3.3. Risk of Bias Assessment Results

**Figure 1:**
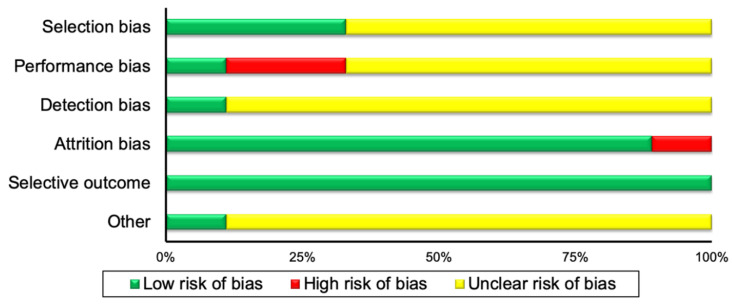
Summary of risk of bias assessment for randomized controlled trials.

**Figure 2:**
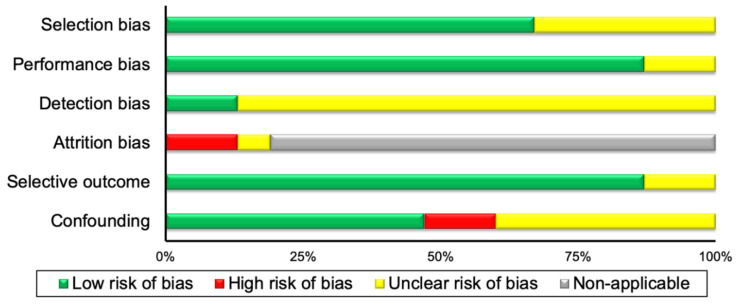
Summary of risk of bias assessment for observational trials.

A summary of the risk of bias assessment of both the included RCTs and observational studies, is illustrated in Figure 1 and Figure 2, respectively. A detailed description of the risk of bias assessment for all the included studies in the current systematic review can be found in Appendix A.

### 3.4. Qualitative Data Synthesis

Food consumption may impact human BAT activity. For example, patients who consumed a high fat, low carbohydrate and protein-permitted diet displayed lower BAT activity (assessed via PET/CT; *p* < 0.0002) and blood glucose levels (*p* < 0.001) compared to patients who followed a fasting protocol [46]. Moreover, in patients with cardiovascular disease, a 12-month supplementation of aged garlic extract (250 mg) plus vitamin-B12 (100 µg), folic-acid (300 µg), vitamin-B6 (12.5 mg) and L-arginine (100 mg) increased epicardial BAT and temperature-rebound in the fingertip compared to placebo supplementation (*p* < 0.05) [40]. A CSS reported a positive association between supraclavicular region temperature with vegetable and protein consumption in females, but not in males, aged 8.5–11.8 years [41]. The latter outcome, however, should be treated with caution, given that skin temperature correlation with BAT activity in humans is relatively low (R^2^ = 0.18–0.23) [60]. Collectively, these limited findings are difficult to incorporate into a firm conclusion regarding the effects of food consumption on human BAT activity.

With a focus on UCP1, in a CSS, there was no association between dietary habits with UCP1 in subcutaneous white adipose tissue in healthy participants (*p* > 0.05) [49]. In another SGDS, an 8-week extremely low-calorie diet decreased browning formation of subcutaneous white adipose tissue in obese individuals, as measured by UCP1 mRNA [59]. This outcome supports previous evidence in animals suggesting that diet has no effect on UCP1 in white adipose tissue [61,62]. In a SGDS, healthy young lean women who were carriers of the UCP1 polymorphism −3826G allele, displayed reduced weight loss after a 2-week hypo-energetic diet consisting of approximately 30% reduction in energy intake [48]. These data support no favorable influence of diet on UCP1 in white adipose tissue and that the UCP1 A−3826G polymorphism may inhibit thermogenic activation of fat in humans. This polymorphism could potentially result in an inhibition of weight loss.

With the consideration of BAT activity in relation to DIT, in a CSS, participants with positive BAT activity (SUV > 2) displayed higher DIT than participants with negative BAT activity (SUV < 2), following the consumption of a meal comprised of 15% protein, 25% fat, and 60% carbohydrate [30]. Similarly, in a SGDS study, ingestion of 9 mg of capsinoids increased postprandial REE more in BAT positive participants (SUV > 2) than in BAT negative participants (SUV < 2)—15.2 ± 2.6 kJ/h against 1.7 ± 3.8 kJ/h, respectively [58]. This evidence supports that higher BAT activity may indicate higher DIT.

The short-term effect of diet on DIT in two SGDS showed that DIT was higher in women with high energy intake compared to women with low energy intake (*p* < 0.05) [55], while DIT was found to be reduced in obese women (8.7 ± 0.8%) compared to non-obese controls (14.8 ± 1.1%) [56]. Moreover, DIT was higher after high carbohydrate meals compared to high fat meals, while red pepper supplementation to either the carbohydrate or high fat meals further increased DIT, particularly after the high fat meals, in healthy women [51]. In a CT, lean healthy women significantly increased DIT (0.79 ± 0.02 to 0.90 ± 0.02 kcal/min, *p* = 0.01) after a meal of 480 kcal (55% carbohydrate, 15% protein, 30% fat) compared to their obese counterparts [54]. Similarly, lean healthy women significantly increased DIT (5574.7 ± 221.2 to 6114.7 ± 239 kJ/day; *p* < 0.01) in response to capsaicin supplementation (3 mg) compared to obese women [53]. Finally, a RCT showed that the thermic effect of food was higher in lean compared to obese young males (*p* = 0.01) after a high-carbohydrate meal (70% carbohydrate, 20% fat and 10% protein) [50]. This evidence suggests that high energy intake, particularly high carbohydrate intake, a lean phenotype, red pepper and capsaicin supplementation are factors that may increase DIT in humans.

### 3.5. Meta-Analysis Outcomes

**Figure 3:**
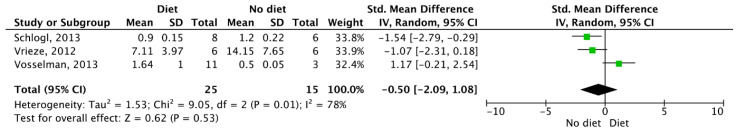
The acute effects of a single meal on human brown adipose tissue activity. SD: Standard deviation, 95%. CI: Confidence interval.

**Figure 4:**
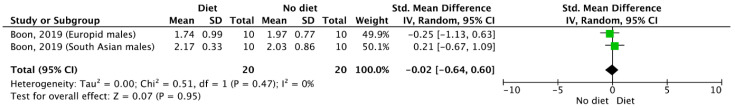
The chronic effects of diet on human brown adipose tissue activity. SD: Standard deviation, 95%. CI: Confidence interval.

Three studies [29,31,45] provided data for a meta-analysis regarding the acute effects of a single meal on BAT activity. These studies showed no mean differences in SUV of BAT (*p* > 0.05) following a high calorie (1622 ± 222 kcal) carbohydrate rich meal [31], a standard (545 kcal) meal [29] and during 200% overfeeding [45] (Figure 3). Finally, one study [42] offered data for a meta-analysis from two different groups of participants, investigating the effects of a 6-week supplementation of L-Arginine on BAT activity, which showed no mean differences in SUV of BAT (*p* > 0.05; Figure 4).

**Figure 5:**
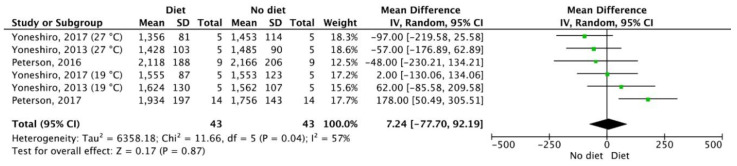
The chronic effects of diet and supplements on resting energy expenditure. SD: Standard deviation, 95%. CI: Confidence interval.

**Figure 6:**
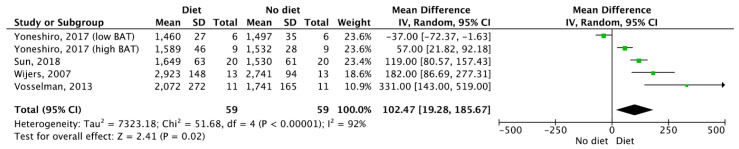
The acute effects of diet and supplements on resting energy expenditure. SD: Standard deviation, 95%. CI: Confidence interval.

Regarding DIT, four studies offered data for a meta-analysis investigating the chronic effect of diet on DIT. This meta-analysis revealed no effect of either 8 weeks [32] or 4 weeks [57] of overfeeding and 6 weeks of capsinoid supplementation [43] as well as 5 weeks of catechin supplementation [44] on REE (*p* > 0.05; Figure 5). Furthermore, four studies provided data for a meta-analysis regarding the acute effects of capsinoid [47], catechin [44] supplementation and a high calorie (1622 ± 222 kcal) carbohydrate rich meal [31] as well as a meal comprised of 47%, 38% and 15% energy from carbohydrate, fat and protein, respectively [52]. This meta-analysis revealed a mean difference in REE between diet and no diet (Z = 2.41, *p* = 0.02; mean difference = 102.47 (95% CI = 19.28–185.67); Figure 6).

## 4. Discussion

The aim of the current systematic review was to explore whether nutritional factors and/or diet affects human BAT activity. We identified 24 eligible studies, of which 10 used PET/CT, three used UCP1 assessments and two used skin temperatures to assess the effects of nutrition on BAT activity. Finally, 9 studies used REE measurements to determine DIT, which involves BAT to increase body temperature [4].

### 4.1. Overall Completeness and Applicability of Evidence

Our meta-analysis confirms that human BAT activity is not increased in response to both a single meal/supplement and long-term dietary habits and/or supplementation. Similarly, we found no chronic effect on REE in response to diet and/or long-term supplementation. We only found that REE is increased in response to a single meal and/or an acute food supplementation. Taken together, this evidence indicates that BAT has no or minor role in DIT following a single meal and/or acute supplementation. Even though the mechanism of the thermic effect of food is not fully defined [4], it has been previously suggested that a single meal can stimulate BAT by interacting with centers in the brain via blood-borne substances (e.g., glucose, insulin, choleocystokinin, enterostatin) [4]. This mechanism acutely increases body temperature in response to a meal. Indeed, plasma secretin levels are increased by a single meal and are positively correlated with postprandial oxygen consumption and fatty acid uptake rates in human BAT [63]. Moreover, human REE is increased in response to a single mixed meal, while oxygen consumption and blood flow in BAT are ameliorated [64]. The repeated single meals, however, do not seem to increase BAT activity past a certain point [4]. Practical implications of these findings suggest that food consumption does not continuously increase BAT activity and, thus, does not seem to exert a chronic influence on BAT activity and related health outcomes. Furthermore, overfeeding leads to increased leptin secretion and impaired interaction with its receptor, which may lead to inadequate communication with the hypothalamus [65]. This may lead to greater fat storage due to increased food intake that may not be countered by increased BAT activity [4]. Indeed, it is inversely associated with BAT activity [1,15].

Our qualitative data synthesis showed that DIT was higher in individuals with positive BAT (SUV > 2) than in individuals with negative BAT (SUV < 2). Considering that increased BAT activity will result in increased UCP1 activity, the latter finding can be explained by the contention that DIT may be influenced by UCP1 activity, while it does not occur as efficiently when UCP1 is absent [26]. However, although DIT was found to be lower in obese than in non-obese in the current systematic review, we were unable to link UCP1 with diet in humans. Similarly, we found little or no evidence to provide an overview of specific diets or supplements that could affect BAT activity in humans. As such, the link between UCP1 with human BAT in response to food consumption and the effects of certain diets/supplementations on human BAT remains to be elucidated.

**Figure 7:**
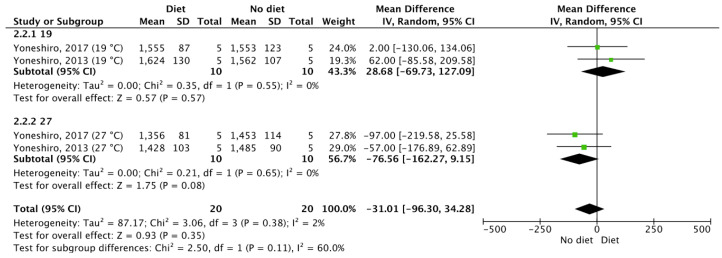
The acute effects of diet and supplements on resting energy expenditure, with regard to ambient temperature. SD: Standard deviation, 95%. CI: Confidence interval.

The current systematic review raised questions regarding the contention that BAT activity in response to food consumption exerts beneficial effects on human health, and therefore, perhaps other factors should also be investigated. For example, leptin may be considered as an index to measure BAT activity and DIT, due to its major homeostatic role in food intake and metabolism [66]. Furthermore, obesity and total fat mass should also be taken into consideration when measuring BAT activity in response to food, due to the positive association between leptin and body fat stores [67] and, as mentioned above, the negative association between obesity and BAT activity [1,15]. It should be noted, however, that leptin is not considered as a thermogenic hormone [68,69]. We have also noticed in one of our meta-analysis (Figure 4) that the lower the environmental temperature, the higher the REE in response to chronic food consumption. In this regard, we performed a subgroup meta-analysis to test whether these differences are significant. We found no differences in REE between measurements in 19° Celsius and 27° Celsius (*p* > 0.05, Figure 7). Even though in this case we found no differences in REE, the higher REE in the colder environments may be explained by the higher BAT activity due to cold exposure [2,70] and not due to food consumption. As such, the environmental temperature should be also considered in measurements of BAT activity in response to food or REE measurements to assess DIT. Finally, the strength of the evidence that the current systematic review provides could be treated with confidence, given that the risk of bias of the included publications is mostly low and unclear in all risk of bias categories (Figure 1 and Figure 2).

### 4.2. Strengths and Limitations

In the current systematic review, we used appropriate algorithms with standardized indexing terms to search the selected databases. Standardized indexing terms may retrieve publications that use alternative words to describe the same concept beyond the one that may be contained in the words of the titles and abstracts [39]. We also used a previous methodology to conduct the systematic review [71,72,73], while we used well-established tools to assess the quality of the included studies [34,35]. Furthermore, to reduce bias, two investigators worked independently on the screening of the included studies for eligibility, risk of bias assessment and data extraction, while we have not excluded studies based on language.

Our systematic review also displays limitations. We used only peer-reviewed literature and did not include grey literature, which may incorporate a publication bias. We did not include grey literature, given the availability of peer-reviewed sources [39]. Moreover, the evidence we have used to synthesize data regarding human BAT may display methodological limitations that may have influenced these measurements [74]. Another limitation is that the number of the available eligible publications was relatively small, while the evidence was disparate. This heterogeneity of the available evidence may have not allowed a vigorous data synthesis.

Our systematic review outcome agrees with a previous similar systematic review [75] suggesting that dietary components may increase human BAT activity; however, the available evidence does not fully support this hypothesis.

## 5. Conclusions

The available evidence revealed that human BAT activity is not significantly affected by nutrition/diet. While REE is increased in response to a single meal, it is unlikely that this is linked with increased BAT activity. Although this conclusion is based on meta-analyses of previous data, the available evidence was limited, and therefore, the meta-analyses outcomes should be treated with caution. Finally, BAT activity assessments in response to the chronic effect of food should be considered along with other factors, such as body composition and environmental temperature. Thus, future randomized controlled trials that consider confounding factors to human BAT activity in response to diet are needed.

## Figures and Tables

**Figure A1:**
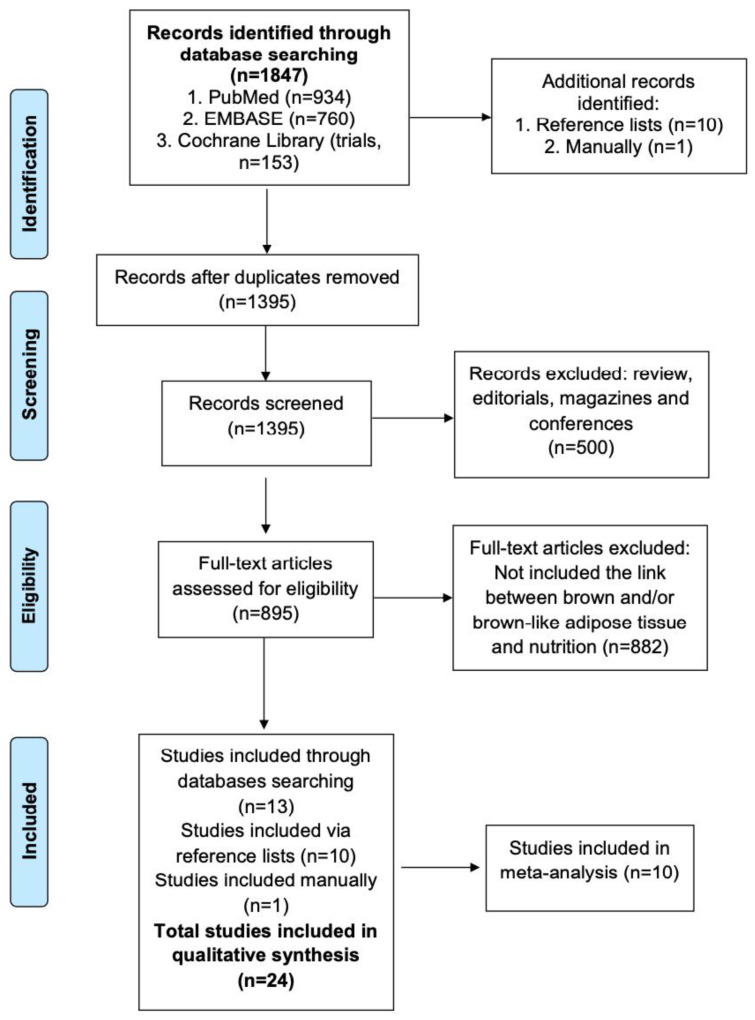
PRISMA flowchart.

## Appendix A

**Search Algorithm in PubMed:**

(((diet*[Title/Abstract]) OR (diet-induced thermogenesis[Title/Abstract]) OR (nutrition*[Title/Abstract]) OR (fasting[Title/Abstract]) OR (high-fat diet[Title/Abstract]) OR (macronutrients[Title/Abstract]) OR (micronutrients[Title/Abstract])) AND ((brown adipose tissue[Title/Abstract]) OR (brown fat[Title/Abstract]) OR (brown-like adipose tissue[Title/Abstract]) OR (beige[Title/Abstract]) OR (brown-like fat[Title/Abstract]) OR (brown adipose-like phenotype[Title/Abstract]) OR (browning process[Title/Abstract]) OR (browning formation[Title/Abstract]))) NOT ((animals[MeSH Terms]) NOT (humans[MeSH Terms]))

**Risk of Bias Assessment Results:**

From the included RCT, 33% displayed low and 67% displayed unclear risk of bias in the selection bias. In performance bias, 22% showed high, 11% displayed low and 67% showed unclear risk of bias. In detection bias, 11% of the RCT showed low and 89% displayed unclear risk of bias, while in attrition bias 11% showed high and 89% low risk of bias. All RCT in selective outcome showed low risk of bias, while in other bias 11% of the RCT displayed high and 89% low risk of bias. Regarding the observational included studies, 33% showed unclear and 67% displayed low risk of bias in selection bias, while in performance bias 13% of studies showed unclear and 87% low risk of bias. In detection bias, 13% of the studies showed unclear and 87% displayed low risk of bias, while in attrition bias 13% showed unclear, 26% showed high and 61% not applicable risk of bias. In selective bias, 26% of the studies displayed unclear and 74% low risk of bias, while in confounding bias, 26% showed high, 40% displayed unclear and 34% showed low risk of bias. The risk of bias assessment for each one of the included studies in the systematic review can be found in the tables below.

**Table A1:** Risk of bias assessment for randomized controlled trials.

First Author	Random Sequence Generation	Allocation Concealment	Blinding of Participants and Researchers	Blinding of Outcome Assessment	Incomplete Outcome Data	Selective Reporting	Other Bias
	Selection		Performance	Detection	Attrition	Reporting	Other
Ahmadi, 2013	**?**	**?**	**?**	**?**	**-**	**+**	**+**
Boon, 2019	**?**	**+**	**+**	**+**	**+**	**+**	**+**
Nagai, 2005	**+**	**?**	**?**	**?**	**+**	**+**	**+**
Schlogl, 2013	**?**	**?**	**?**	**?**	**+**	**+**	**+**
Vrieze, 2012	**?**	**?**	**?**	**?**	**+**	**+**	**+**
Wijers, 2007	**?**	**?**	**?**	**?**	**+**	**+**	**+**
Yoshioka, 1998	**?**	**?**	**?**	**?**	**+**	**+**	**-**
Yoneshiro, 2013	**?**	**?**	**-**	**?**	**+**	**+**	**+**
Yoneshiro, 2017	**+**	**?**	**-**	**?**	**+**	**+**	**+**

**Key: +**: low risk of bias; **-**: high risk of bias; **?**: unclear risk of bias.

**Table A2:** Risk of bias assessment for observational trials.

First Author	Selection	Performance	Detection	Attrition	Reporting	Confounding
Barquissau, 2018	**?**	**+**	**?**	**N**	**+**	**?**
Dinas, 2017	**+**	**+**	**+**	**N**	**+**	**+**
Hibi, 2016	**+**	**+**	**?**	**N**	**+**	**+**
Matsumoto, 2001	**+**	**+**	**?**	**-**	**+**	**+**
Matsumoto, 2000	**+**	**+**	**?**	**-**	**+**	**-**
Nagai, 2011	**?**	**?**	**?**	**N**	**+**	**+**
Peterson, 2017	**?**	**+**	**?**	**N**	**+**	**?**
Peterson, 2016	**+**	**+**	**?**	**N**	**+**	**?**
Robinson, 2019	**+**	**+**	**?**	**N**	**+**	**-**
Schutz, 1984	**?**	**+**	**?**	**N**	**+**	**+**
Sun, 2018	**+**	**+**	**?**	**?**	**?**	**+**
Vosselman, 2013	**+**	**+**	**?**	**N**	**+**	**?**
Weststrate, 1993	**?**	**+**	**?**	**N**	**?**	**+**
Williams, 2008	**+**	**?**	**?**	**N**	**+**	**?**
Yoneshiro, 2012	**+**	**+**	**+**	**N**	**+**	**?**

**Key: +**: low risk of bias; **-**: high risk of bias; **?**: unclear risk of bias; **N**: not applicable.

**Table A3:** Characteristics of the included studies.

Study	Design	Participants Characteristics	Intervention	Main Outcome
**Ahmadi, 2013**	RCT	65 (n = 33 AGE-S (aged garlic extract plus supplement); n = 32 placebo) participants 40–79 yr. and free from clinical coronary artery disease; 51 M and 14 F.	Daily capsule of placebo or AGE-S (aged garlic-extract (250 mg), vitamin-B12 (100 µg), folic-acid (300 µg), vitamin-B6 (12.5 mg) and L-arginine (100 mg)) for 12-months.	AGE-S participants showed higher brown epicardial adipose tissue (bEAT) (AGE-S: 43.4 ± 15.9; placebo: 33.7 ± 13.89) and temperature-rebound when compared to placebo (*p* < 0.05) after 12-months.
**Barquissau, 2018**	SGDS	289 obese males (n = 101, BMI: 33.7 ± 4.6 kg/m^2^, age: 43.4 ± 5.9 yr.) and females (n = 188, BMI: 34.5 ± 4.6 kg/m^2^, age: 41.7 ± 6.4 yr.).	Dietary intervention performed in two phases. Phase one: 8-week very low-calorie diet. Phase two: 6-month weight maintenance period.	Decreased browning of subcutaneous abdominal white adipose tissue was reported after the very low-calorie diet. Changes observed in body fat and insulin resistance were not dependent on changes in brown and beige fat markers.
**Boon, 2019**	RCT	10 prediabetic overweight Dutch South Asian males, (age: 46.5 ± 2.8 yr., BMI: 30.1 ± 1.1 kg/m^2^) and 10 prediabetic Dutch males of European decent (age 47.5 ± 2.0 yr., BMI: 30.7 ± 1.2 kg/m^2^).	Participants ingested either L-arginine (9 g/day) or placebo tablets for 6 weeks followed by a 4-week washout period.	Six weeks of L-arginine supplementation did not influence body weight, BMI, fat mass or lean mass in either the Dutch South Asian group or the Dutch males of European decent. The mean and maximum BAT activity values (expressed as SUV) did not differ between groups and were not influenced by L-arginine treatment.
**Dinas, 2017**	CSS	32 healthy, non-smoking males (age: 36.1 ± 7.4 yr., BMI: 27.1 ± 4.6 kg/m^2^) free from chronic disease.	Diet recalls were retrieved from two weekdays and one weekend day (randomly selected) to assess energy/nutrient intake during the week prior to measurements. Measurements included body composition, REE and a subcutaneous fat biopsy following a 12-h fast and after refraining from exercise, alcohol and passive smoking for 72 h. Fat biopsy samples were used to assess UCP1, PGC-1α, PPARα and PPARγ mRNA expression.	Diet was not associated with browning formation markers of subcutaneous adipose tissue in healthy men. UCP1 mRNA in white adipose tissue was not linked to body weight or body composition. Activation of the PGC-1α, PPARα and PPARγ genes could collectively indicate browning formation of white adipose tissue through increased UCP1 expression.
**Hibi, 2016**	CSS	21 healthy males between 20–50 yr. with a BMI of 18.0–24.9 kg/m^2^. Subjects were divided into BAT-positive (n = 13) and BAT-negative (n = 8) groups according to F-FDG-PET/CT findings.	A prescribed meal was given at 18:00 h before energy metabolism was measured. Subjects ate the same meal/quantity at 09:00 h (breakfast), 14:00 h (lunch) and 19:00 (dinner) and were instructed to drink water ad libitum. The three meals were comprised. BAT activity was measured using PET/CT.	Diet induced thermogenesis and fat utilization were higher in BAT-positive subjects than in the BAT-negative subjects. These findings suggest that brown adipose tissue may have a physiologic role in energy metabolism. Mean SUV max was 8.5 ± 4.8 in the BAT-positive group and 1.1 ± 0.4 in the BAT-negative group (*p* < 0.001).
**Matsumoto, 2000**	CT	8 lean (age: 19.6 ± 0.3 yr., BMI: 21.0 ± 0.6 kg/m^2^) and 8 obese (age: 20.1 ± 0.4 yr., BMI: 28.8 ± 1.0 kg/m^2^) females.	Participants were served rice with spicy yellow curry sauce containing 3 mg of capsaicin over a ten-minute period. The experimental meal was composed of 60% carbohydrate, 30% fat and 10% protein. The energy content of the meal was 2016 kJ. DIT was assessed via energy expenditure measurements.	The lean females experienced an increase in energy expenditure after the meal (5574.7 ± 221.2 to 6114.7 ± 239 kj*day-1; *p* < 0.01). No postprandial changes were observed in the obese group (6191.8 ± 274.3 to 6531.8 ± 337.3 kj*day-1).
**Matsumoto, 2001**	CT	8 obese (age: 20.0 ± 0.3 yr., BMI: 29.0 ± 1.0 kg/m^2^) and 8 non-obese (age: 19.8 ± 0.9 yr., BMI: 18.6 ± 0.4 kg/m^2^) females.	Participants consumed a mixed food meal (480 kcal; 55% carbohydrate, 15% protein and 30% fat) over a 5-min period. DIT was assessed via energy expenditure measurements.	Energy expenditure was increased in the non-obese group (0.79 ± 0.02 to 0.90 ± 0.02 kcal/min, *p* < 0.01) and the obese group (1.00 ± 0.02 to 1.06 ± 0.03 kcal/min, *p* < 0.05) after the mixed-food meal. Magnitude of the increase above the pre-meal condition was greater in the non-obese compared to the obese participants (11.2 ± 2.3 vs. 6.7 ± 0.8%, *p* < 0.05).
**Nagai, 2005**	RCT	13 healthy, lean (age: 8.8 ± 0.4 yr., BMI: 16.5 ± 0.4 kg/m^2^) and 10 obese (age: 9.2 ± 0.4 yr., BMI: 23.3 ± 0.8 kg/m^2^) boys.	Different menus served on two different days were provided to the two groups. One menu consisted of a high carbohydrate meal (70% carbohydrate, 20% fat and 10% protein) and the other consisted of a high fat meal (20% carbohydrate, 70% fat and 10% protein). Each meal was standardized, 80 kJ per kg of actual body mass (30.9 ± 1.0 kg) in lean boys and 80 kJ per kg of ideal body mass (33.2 ± 1.6 kg) in obese boys. Thermic effect of food (TEF) was assessed via energy expenditure measurements.	The obese group experienced a smaller increase in VO_2_ (lean, 1.25 ± 0.02 L; obese, 1.15 ± 0.20 L; *p* < 0.01) and in VCO_2_ (lean, 1.26 ± 0.03 L; obese, 1.16 ± 0.03 L; *p* <0.05) after the high carbohydrate meal. Following the high fat meal, the net increase in VO_2_ was not different between groups (lean, 1.24 ± 0.03 L; obese, 1.19 ± 0.02 L), but the net increase in VCO_2_ was lower in the obese group compared to the lean group (lean, 1.15 ± 0.03 L; obese, 1.08 ± 0.02 L; *p* < 0.05). Obese boys appeared to experience a diminished TEF response to the high carbohydrate meal.
**Nagai, 2011**	SGDS	17 female volunteers (age: 20–22 yr.) with normal weight and BMI, percent body fat between 25.1–38.5%, free from disease and were not using any treatment known to affect weight loss.	Participants were fed a hypoenergetic diet consisting of a 30% reduction in energy intake during a 2-week energy-restriction period. During the energy-restriction period, participants were restricted to 5.0 MJ (1200 kcal)/d (62% carbohydrate, 19% protein and 19% fat), consisting of 3 isoenergetic, nutritionally balanced meals. During the experimental period, participants were not permitted to consume soft drinks, alcoholic beverages or any food not included in the test meals.	Following the intervention, the G allele participants experienced significantly smaller changes in body weight, BMI and waist circumference compared to the A/A genotype participants. These data suggest that the UCP1 gene −3826 G allele could contribute to smaller weight loss after a short-term, controlled-energy diet in young, lean women.
**Peterson, 2016**	SGDS	9 healthy, non-smoking males (age: 23 ± 3 yr., 23.0 ± 1.8 kg/m^2^). Volunteers consumed less than 3 alcoholic drinks/day, were not currently on medication, had no recent change in body weight (>2 kg in the prior 6 months), had no impaired fasting glucose (>100 mg/dL), did not exercise intensely (>3 times/week) and had no chronic disease.	Participants were exposed to cold for 20 min per day, for five days per week for four weeks. DIT was determined during a 24-h thermic response to one day of 50% overfeeding.	Participants were overfed by 50.2 ± 4.6% at baseline versus 53.1 ± 3.4% post-cold acclimation. 24-h thermic response following overfeeding was similar at baseline (2166 ± 206 kcal/day) and following the four-week cold intervention (2118 ± 188 kcal/day; *p* = 0.15). Cold acclimation did not change the thermic response following overfeeding.
**Peterson, 2017**	SGDS	14 males (age: 24 ± 3 yr., BMI: 24.5 ± 1.6 kg/m^2^).	Participants were overfed by 40% for 8 weeks. The diet composed of 41% carbohydrate, 44% fat and 15% protein. The PBRC Metabolic Kitchen prepared all 21 meals during the 8-week intervention. BAT activity was measured using infrared imaging of the supraclavicular BAT depot.	Metabolic adaptation increased from -0.9 ± 3.9% to 4.7 ± 5.6% (*p* = 0.001). BAT thermal activity remained similar (*p* = 0.81). BAT thermal activity was not associated with the degree of metabolic adaptation (*p* = 0.32) or with the change in body weight (*p* = 0.51).
**Robinson, 2019**	CSS	36 children (16 boys and 20 girls; age: 8.5–11.8 yr.).	A survey on the child’s diet was completed by the parents. Based on the answers, foods were sorted into categories of carbohydrate, dairy, fruit, protein, savory, sweet and vegetable. Infrared thermography of the neck and upper thorax was utilized to examine BAT activity.	BAT thermogenesis may be altered by dietary intake in a sex-specific manner. A correlation between the supraclavicular region temperature and report of vegetable and protein consumption was observed in young girls. After adjustment for multiple testing in the study sample, the relationships were no longer statistically significant. There were no associations between supraclavicular region temperature and food consumption in any category for the young boys. There was no difference in vegetable and protein consumption scores between girls and boys.
**Schlogl, 2013**	RCT	16 healthy women (n = 7, age: 30.7 ± 8.6 yr., BMI 28.0 ± 6.5 kg/m^2^) and men (n = 9, age: 31.1 ± 11.3 yr., BMI: 25.1 ± 4.6 kg/m^2^).	Volunteers followed a weight-maintenance diet composed of 50% carbohydrate, 30% fat and 20% protein. Each participant completed 24-h EE measures, during energy balance, fasting and during 200% over feeding (60% fat, 20% protein, 20% carbohydrate). The first six participants had a second PET/CT after 36 h of fasting to further examine BAT activation at 22 °C. Other participants had a second PET/CT after 24 h of overfeeding at 22 °C but only if they showed cold-induced BAT activity.	Cold-induced BAT activity was seen in 8 of 10 participants after overfeeding. DIT was 280 ± 164 kcal during overfeeding vs 140 ± 116 kcal during energy balance (*p* = 0.009). The mean SUV of BAT (n = 8) after overfeeding (0.9 ± 0.2) compared to cold-induced BAT activity (3.5 ± 0.7) was lower (*p* = 0.001).
**Schutz, 1984**	SGDS	28 females (age: 19–44 yr.) 20 were obese (percent body fat: 38.6 ± 0.7%), and 8 were non-obese (percent body fat: 24.7 ± 0.9%).	For the weight maintenance period, 1–2 weeks before the study, subjects consumed their normal diet. Each subject ate three meals prepared by a dietician including normal “natural” foods (breakfast: bread, butter, marmalade, milk; lunch and dinner: bread, meat or fish, vegetables, dessert). Decaffeinated coffee was served at each meal, and no alcohol was consumed during the experiment. Energy expenditure was measured for 24 h in a respiration chamber.	The thermogenic response to the three meals was found to be low in the obese participants (8.7 ± 0.8%) when compared to the controls’ (14.8 ± 1.1%). The thermogenic response induced by the three meals was negatively correlated with body weight (r = −0.552, *p* < 0.01) and percent body fat (r = −0.613, *p* < 0.001).
**Sun, 2018**	CT	20 healthy males and females (age: 21–35 yr., BMI: 18.5–26.0 kg/m^2^). All participants were healthy, no history of diabetes or cardiovascular disease, smoke or use tobacco. They did not adhere to special diets or take medication known to alter brown adipose tissue metabolism.	PET/CT measurement and whole-body calorimetry were assessed after capsinoid ingestion (12 mg) or cold exposure (~14 °C) in a crossover design.	Capsinoid ingestion did not result in detectable BAT activation, as all participants in each trial stayed at or below the level of baseline-detectable activity assessed during the PET/CT scan. The results showed that ingestion of capsinoids led to a bigger increase in energy expenditure (10%) in BAT-positive participants than in BAT-negative participants (5%).
**Vosselman, 2013**	SGDS	11 lean males (age: 23.6 ± 2.1 yr., BMI: 22.4 ± 2.1 kg/m^2^).	Participants consumed a high calorie and carbohydrate rich meal (1622 ± 222 kcal; 78% carbohydrate, 12% protein, 10% fat). BAT activity was assessed by (18F) FDG-PET/CT following consumption of the meal. BAT assessed during 2 h of cold exposure served as a positive control. Energy expenditure was assessed via indirect calorimetry.	BAT activity following the meal was lower compared to cold-induced BAT activity. There was no direct relationship between BAT activity and DIT.
**Vrieze, 2012**	RCT	10 healthy, lean males (age: 18–32 yr., BMI 20–24 kg/m^2^).	Each volunteer underwent two PET/CT scans two weeks apart. The first scan was completed after an overnight fast and the second scan was completed after an overnight fast with a standardized meal consumed 90 min beforehand. The meal was a chicken-bacon sandwich and 200 mL of whole milk (545 kcal), containing 34g of fat, 37g or carbohydrates and 23g of protein.	BAT activity was observed in 6 of 10 volunteers. All subjects with BAT activity had higher SUVmax in the fasted state (median, 13.1 g/mL; range, 6.1–27.6 g/mL) than in the post-meal state (median, 6.8 g/mL; range 2.1–13.4 g/mL) (*p* = 0.03). Cold activated BAT in humans is more distinct during fasting. Meal-induced insulin secretion could explain the postprandial decrease in BAT.
**Weststrate, 1993**	SGDS	49 non-obese males and 54 women (22 non-obese and 32 obese).	DIT was assessed using a ventilated-hood system. In males: Study 1 tested the thermic effect of alcohol. Study 2 examined the impact of palatability on DIT. Study 3 examined a 2-week dietary intervention on individual energy metabolism. In females: Studies 1 and 2, were similar to the studies conducted in males. Studies 3 and 4 examined the effect of the ovular phase of the menstrual cycle on RMR and DIT. Study 5 looked at the effect of body-fat distribution in obesity on energy metabolism. Study 6 studied the effect of body-fat distribution on weight loss and energy metabolism in obese women. DIET: nine subjects, four males and five females, followed a 2-week diet. Food was provided in a 4-day rotating menu with minor differences in energy content (CV<5%) and nutrient composition (CV protein, fat < 10%, CV carbohydrates < 15%) between the four menus. Mean energy intake was 9.3 ± 0.5 MJ/d.	Variation in DIT was not changed when the diet was controlled. Total DIT values were significantly higher (*p* < 0.05) in women with higher energy intakes. No significant differences were found between both groups in DIT, when expressed as a percentage of the energy content of the test meal, 6.62 ± 0.29% in the group with high energy intake and 6.24 ± 0.50% in the group with low energy intake.
**Wijers, 2007**	RCT	13 lean males (age 22.8 ± 1.7 yr., BMI: 22.96 ± 0.90 kg/m^2^).	Participants underwent three different experimental conditions in a respiration chamber once for 36 h (control meal) and twice for 84 h (overfeeding at 16 °C and at 22 °C).	Overfeeding showed significant increases in EE (0.77 MJ/d, *p* < 0.001). The increase in EE during overfeeding was significantly related to the increase in EE during mild cold exposure (r = 0.63; *p* < 0.05). This suggests that both overfeeding-induced and mild cold-induced adaptive thermogenesis share similar mechanisms.
**Williams, 2008**	CSS	Fasting protocol (n = 1229; age: 58 ± 16 yr., male 58%, female 48%). High fat, low carbohydrate, protein permitted diet (n = 741; age: 58 ± 16 yr., male 53%, female 47%).	Consumption of a high fat, very low carbohydrate, protein permitted diet. Brown adipose tissue activity was assessed via PET/CT measurements.	The results showed a difference between the fasting and high fat, low carbohydrate group in blood glucose and frequency of FDG uptake by hypermetabolic brown adipose tissue. Participants who consumed the high-fat diet experienced a significant reduction in the frequency of hypermetabolic BAT uptake (*p* < 0.0002) and had lower blood glucose levels (*p* < 0.001).
**Yoneshiro, 2012**	SGDS	18 healthy males (age: 20–32 yr.) separated into BAT-positive (n = 10) and BAT-negative (n = 8) groups after FDG uptake was assessed.	2 h of cold exposure while wearing light clothing after oral ingestion of capsinoids (9 mg). Brown adipose tissue was assessed using PET/CT measurements.	Energy expenditure increased by 15.2 ± 2.6 kJ/h in 1 h in the BAT-positive group and by 1.7 ± 3.8 kJ/h in the BAT-negative group following the intervention (*p* < 0.01). Significant effects of time (*p* < 0.001), capsinoids x BAT (*p* < 0.05) and time x capsinoids x BAT (*p* < 0.05) were found. The BAT-positive group showed an increase in energy expenditure. This increase was significant, 0.5–2 h after capsinoids treatment (maximal increase of 502 ± 81 kJ/d at 1 h). Energy expenditure slightly changed after placebo ingestion.
**Yoneshiro, 2013**	RCT	51 healthy males (age: 22.4 ± 0.5 yr., BMI: 22.0 ± 0.4 kg/m^2^). Only 10 males were selected to complete the capsinoid test, with low or undetectable BAT activity.	The 10 males ingested capsules containing 9 or 0 mg (placebo) capsinoids every day for 6 weeks. BAT was assessed via PET/CT measurements.	Daily ingestion of capsinoids (9 mg) and cold exposure can brown adipose tissue even among individuals who have lost active brown adipose tissue.
**Yoneshiro, 2017**	RCT	15 healthy males participated in the acute catechin trial. 10 healthy males who showed low or no BAT activity participated in the chronic catechin trial.	The experiment consisted of a single ingestion of a beverage containing 615 mg catechin and 77 mg of caffeine in 350 mL. The control beverage contained 0 mg of catechin and 81 mg of caffeine. Ingestion occurred for 5 weeks, twice per day. Participants maintained their daily lifestyle, including dietary intake and physical activity during the experimental period. BAT activity was assessed via PET/CT measurements.	Ingestion of the catechin beverage increased energy expenditure in 9 participants who had active BAT (mean ± SEM: +15.24 ± 1.48 kcal, *p* = 0.01) but not in 6 participants who had inactive BAT (mean ± SEM: +3.42 ± 2.68 kcal). The placebo beverage containing 82 mg of caffeine produced a smaller and comparative EE response. The recruitment of BAT in humans possibly led to increased EE levels and non-shivering CIT through the tea catechin with caffeine.
**Yoshioka, 1998**	RCT	13 healthy females (age: 25.8 ± 2.8 yr., body weight: 54.2 ± 6.4 kg).	Women consumed a standardized meal before beginning the experiment with a standardized breakfast. The breakfast fell under one of the following four conditions: high fat meal, high fat and red-pepper (10 g) meal, high calorie meal or high calorie and red-pepper meal. The experimental meals consisted of a stir fry of rice, scallops, shrimps, bacon, green peppers, green peas, onions and tomatoes. DIT was assessed via energy expenditure measurements.	Diet induced thermogenesis was significantly higher after the high calorie meals than after the high fat meal. The addition of red pepper to the meals significantly increased diet-induced thermogenesis and lipid oxidation, especially in the high fat meal.

**Key**: RCT = Randomized controlled trial; AGE-S = Aged garlic extract with supplement; SGDS = Single group design study; FDG-PET/CT = Fluorodeoxyglucose-positron emission tomography/computed tomography; BMI = Body mass index; BAT = Brown adipose tissue; CSS = Cross-sectional study; DIT = Diet-induced thermogenesis; SUV = Standardized uptake value; UCP1 = Uncoupling protein one; CT = Controlled trial; EE = Energy expenditure; SEM = Standard error of the mean.

**Table A4:** The study’s Preferred Reporting Items for Systematic Reviews and Meta-Analyses (PRISMA) checklist.

Section/Topic	Identification Number	Checklist Item	Reported on Page
**TITLE**			
Title	1	Identify the report as a systematic review, meta-analysis or both.	1
**ABSTRACT**			
Structured summary	2	Provide a structured summary including, as applicable, background; objectives; data sources; study eligibility criteria, participants and interventions; study appraisal and synthesis methods; results; limitations; conclusions and implications of key findings; systematic review registration number.	1
**INTRODUCTION**			
Rationale	3	Describe the rationale for the review in the context of what is already known.	1–2
Objectives	4	Provide an explicit statement of questions being addressed with reference to participants, interventions, comparisons, outcomes and study design (PICOS).	2
**METHODS**			
Protocol and registration	5	Indicate if a review protocol exists, if and where it can be accessed (e.g., Web address) and, if available, provide registration information including registration number.	2
Eligibility criteria	6	Specify study characteristics (e.g., PICOS, length of follow-up) and report characteristics (e.g., years considered, language, publication status) used as criteria for eligibility, giving rationale.	2–3
Information sources	7	Describe all information sources (e.g., databases with dates of coverage, contact with study authors to identify additional studies) in the search and date last searched.	2
Search	8	Present full electronic search strategy for at least one database, including any limits used, such that it could be repeated.	2
Study selection	9	State the process for selecting studies (i.e., screening, eligibility, included in systematic review and, if applicable, included in the meta-analysis).	2–3
Data collection process	10	Describe method of data extraction from reports (e.g., piloted forms, independently, in duplicate) and any processes for obtaining and confirming data from investigators.	3
Data items	11	List and define all variables for which data were sought (e.g., PICOS, funding sources) and any assumptions and simplifications made.	3
Risk of bias in individual studies	12	Describe methods used for assessing risk of bias of individual studies (including specification of whether this was done at the study or outcome level) and how this information is to be used in any data synthesis.	3
Summary measures	13	State the principal summary measures (e.g., risk ratio, difference in means).	3
Synthesis of results	14	Describe the methods of handling data and combining results of studies, if done, including measures of consistency (e.g., I^2^) for each meta-analysis.	3
Risk of bias across studies	15	Specify any assessment of risk of bias that may affect the cumulative evidence (e.g., publication bias, selective reporting within studies).	3
Additional analyses	16	Describe methods of additional analyses (e.g., sensitivity or subgroup analyses, meta-regression), if done, indicating which were pre-specified.	N/A
**RESULTS**			
Study selection	17	Give numbers of studies screened, assessed for eligibility and included in the review, with reasons for exclusions at each stage, ideally with a flow diagram.	3–4
Study characteristics	18	For each study, present characteristics for which data were extracted (e.g., study size, PICOS, follow-up period) and provide the citations.	3–4
Risk of bias within studies	19	Present data on risk of bias of each study and, if available, any outcome level assessment (see item 12).	4 and Appendix
Results of individual studies	20	For all outcomes considered (benefits or harms), present, for each study, (a) simple summary data for each intervention group and (b) effect estimates and confidence intervals, ideally with a forest plot.	4–6
Synthesis of results	21	Present the main results of the review. If meta-analyses are done, include for each confidence intervals and measures of consistency	3–6
Risk of bias across studies	22	Present results of any assessment of risk of bias across studies (see Item 15).	4 and Appendix
Additional analysis	23	Give results of additional analyses, if done (e.g., sensitivity or subgroup analyses, meta-regression (see Item 16)).	N/A
**DISCUSSION**			
Summary of evidence	24	Summarize the main findings including the strength of evidence for each main outcome; consider their relevance to key groups (e.g., healthcare providers, users and policy makers).	6–7
Limitations	25	Discuss limitations at study and outcome level (e.g., risk of bias) and at review-level (e.g., incomplete retrieval of identified research, reporting bias).	8
Conclusions	26	Provide a general interpretation of the results in the context of other evidence and implications for future research.	7–8
**FUNDING**			
Funding	27	Describe sources of funding for the systematic review and other support (e.g., supply of data); role of funders for the systematic review.	N/A
